# Load Transfer in Tibial Intramedullary Nailing: Effects of Fracture Level, Screw Configuration and Nail‐Canal Clearance

**DOI:** 10.1002/jor.70102

**Published:** 2025-12-16

**Authors:** José L. M. Thiesen, Rafael Prado, Kodi E. Kojima, Tóride S. Celegatti, Paulo de Tarso R. Mendonça, Carlos R. M. Roesler, Eduardo A. Fancello

**Affiliations:** ^1^ GRANTE ‐ Mechanical and Analysis Design Group, Department of Mechanical Engineering Federal University of Santa Catarina (UFSC) Florianópolis SC Brazil; ^2^ LEBm ‐ Biomechanical Engineering Laboratory, Department of Mechanical Engineering Federal University of Santa Catarina (UFSC) Florianópolis SC Brazil; ^3^ IOT/HCFMUSP ‐ Institute of Orthopedics and Traumatology, Hospital das Clínicas, School of Medicine University of São Paulo (USP) São Paulo SP Brazil; ^4^ Toride IND. COM. LTDA Mogi Mirim SP Brazil

**Keywords:** fracture fixation, fracture healing, interlocking screws, Intramedullary nail, tibial fractures

## Abstract

Intramedullary nails are widely recognized for their high stabilization capacity and are considered the gold standard for tibial fracture treatment. However, hardware failure, especially of distal screws, is frequently reported, especially in cases involving distal fractures. Although various experimental studies explore screw configurations, few quantify the mechanical loads acting on individual screws at different fracture locations. This study numerically evaluates two fixation systems: one with four screws and another with five, including an additional distal screw (DS3). The results show that the fifth screw significantly reduces von Mises stress, especially in distal fractures, though the improvements in overall stability are less pronounced. The mechanical implications on the inclusion of a clearance between the nail and endosteal surface are assessed. The stresses in the locking screws are significantly higher when the reaming clearance is included in the simulation. Despite mechanical advantages, clinical trade‐offs such as increased surgical complexity and radiation exposure must be considered.

## 1 Introduction

In 1940, German surgeon Gerhard Küntscher (1900–1972) introduced the use of hollow stainless‐steel rods, known as intramedullary nails, to stabilize diaphyseal femoral fractures by inserting them into the intramedullary cavity [1]. The early nail designs had a drawback, which was the lack of postoperative stability in complex fractures such as oblique or comminuted fractures. This problem was later overcome with the use of interlocking screws, which provide crucial axial stability to prevent limb shortening, while also enhancing the construct's resistance to bending and torsional forces. Although used for a limited number of cases until the second half of the twentieth century, intramedullary nails (IMN) are now considered the gold standard for the treatment of tibial fractures [2].

However, critical device failures are frequently observed in fractures located in the proximal or distal metaphyseal regions [3]. Several studies have investigated the impact of increasing the number of interlocking screws and varying their configurations on the mechanical stability of the intramedullary nail system. Despite these efforts, there is no consensus in the literature regarding the optimal number of distal screws, nor is there consensus on their quantity or configuration for fractures at different locations (proximal or distal). Although some authors advocate the use of a single screw to reduce the operative time and minimize radiation exposure [4], others recommend the use of two mediolateral distal screws [5] or one mediolateral plus an anteroposterior screw [6]. In addition, the efficacy of using more than two distal screws has also been investigated, incorporating various configurations [7]. The authors of Cunningham et al. [8] investigated the case of long‐distance nailing, and highlighted that increasing the number of distal interlocks improved the stability of the overall system.

Kneifel and Buckley [9] conducted a clinical trial to evaluate the effect of varying the number of distal screws on mechanical failure rates. With one proximal screw used in all patients, the study revealed that constructs with only one distal screw exhibited a higher failure rate than those with two distal screws. In addition, the rate of screw failure increased as patients were subjected to greater weight‐bearing forces during the postoperative period. The authors also noted that patients with higher body weight were more susceptible to distal interlocking screw failure. Guran et al. [10] performed experiments to assess the biomechanical stability of the intramedullary tibial nails at different fracture levels and varying numbers of distal screws. They argue that the use of two distal screws is sufficient fixation, regardless of the fracture level. However, as mentioned by Hutson et al. [11], distal screws are particularly prone to failure as the fracture line moves distally.

Several authors have attempted to investigate further aspects of this problem using the finite element method, which is adequate to evaluate the stresses and strains of the bone‐implant‐screw system [12, 13, 14]. In Raunest et al. [15], the authors have developed a three‐dimensional finite element model of such systems to evaluate the stability and failure risk of the implant. The authors mention that a torque load can induce critical stresses in the distal screws.

Chao et al. [16] performed parametric finite element analyses to evaluate the effect of design factors such as root radius and inner diameter of the interlocking screws on their mechanical strength and fatigue behavior. The boundary conditions and loading conditions were based on the three‐point bending test, which is mentioned as a limitation from the study. Gómez‐Benito et al. [17] compared the biomechanical performance of reamed and unreamed intramedullary nail systems. They reported that the unreamed technique, which employs a smaller‐diameter nail, results in increased stresses on the interlocking screws. In their simulations, however, the clearance between the nail and the endosteal surface of the trabecular bone was assumed to be zero for both techniques. More recently, Tucker et al. [18] investigated the influence of canal–nail clearance on intramedullary femoral nails and demonstrated that greater canal filling reduces peak implant stresses. Although this finding is mechanically favorable, achieving an optimal match between the reamed canal and the nail diameter is clinically improbable, as excessive interference during insertion markedly increases the risk of iatrogenic fracture.

The purpose of the present study is to investigate the role of the tibial fracture position on the mechanical loading of interlocking screws with a particular focus on the distal interlocking screws adjacent to the fracture, which is considered to be the most prone to failure. It is hypothesized that, as the fracture site approaches the distal region, the distal interlocking screws are subjected to an increased resultant moment, which can lead to static and/or cyclic modes of failure such as fatigue. The inclusion of additional distal interlocking screws for fixation is regarded as a safety‐oriented design consideration, with the objective of decreasing the loads to which the distal interlocking screws are subjected. Additionally, the study aims to evaluate the stabilization provided by different screw configurations by analyzing the relative motion between the fracture surfaces. This approach enables the extension of the findings of Chan et al. [19] to other configurations and fracture levels, providing a more comprehensive understanding of the effect of screw placement on fracture stabilization. All simulations in this study consider two scenarios: (i) a full match between the nail diameter and the endosteal surface of the trabecular bone, and (ii) a canal–nail clearance of 1 mm. This approach enables the assessment of the biomechanical consequences of such clearance during the surgical procedure. We hypothesize that increasing the canal–nail gap shifts the majority of the load‐bearing function onto the interlocking screws.

The paper is structured as follows: Section 2 describes the main aspects of the computational model and details of the simulation procedure. Numerical results are presented in Section 3, focusing on the forces, moments and stresses on the screws, as well as the relative displacements between the fracture surfaces. Discussion of these results is presented in Section 4, while final conclusions are presented in Section 5.

## 2 Methods

### 2.1 Geometry Preparation

The geometric model of the left tibia was sourced from the GrabCAD library [20] and adjusted to capture the key anatomical features of the adult human tibia. Cortical and trabecular regions were represented by proportionally scaling the external geometry. A patient‐specific approach using CT‐based Hounsfield units could have been applied, but the scaled model provided a simpler and sufficiently accurate representation for this comparative study.

**Figure 1:**
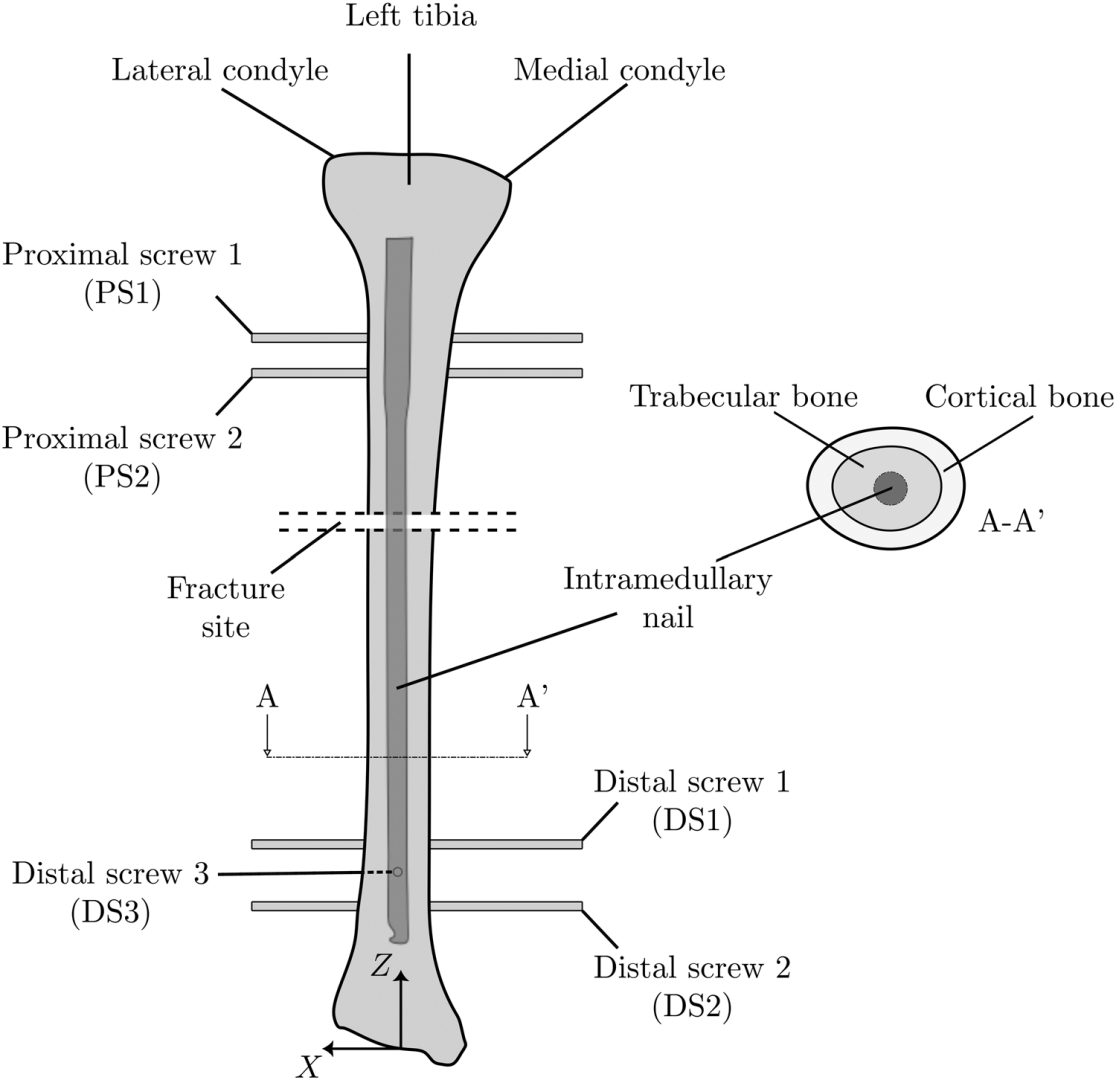
Description of the geometry and all anatomical features modeled in CAD.

**Figure 2:**
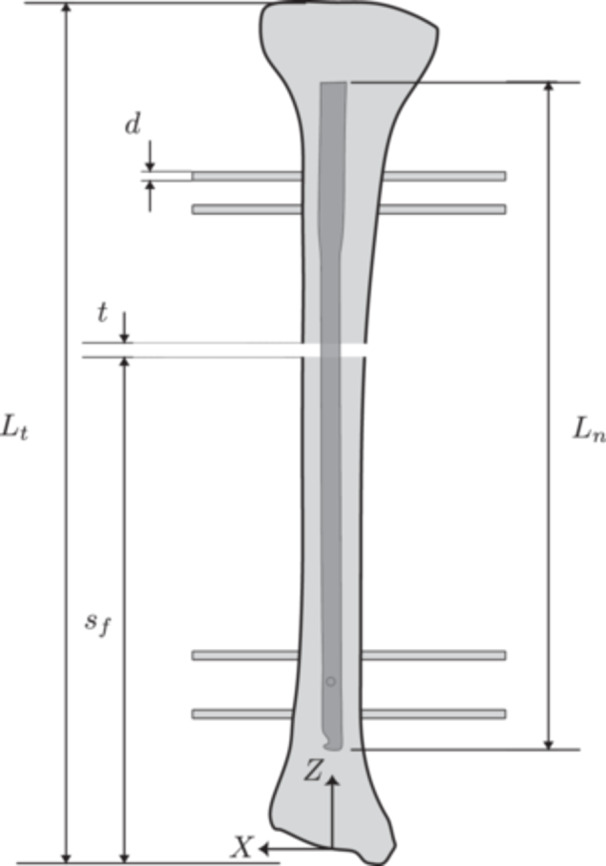
Geometrical parameters of the model.

**Figure 3:**
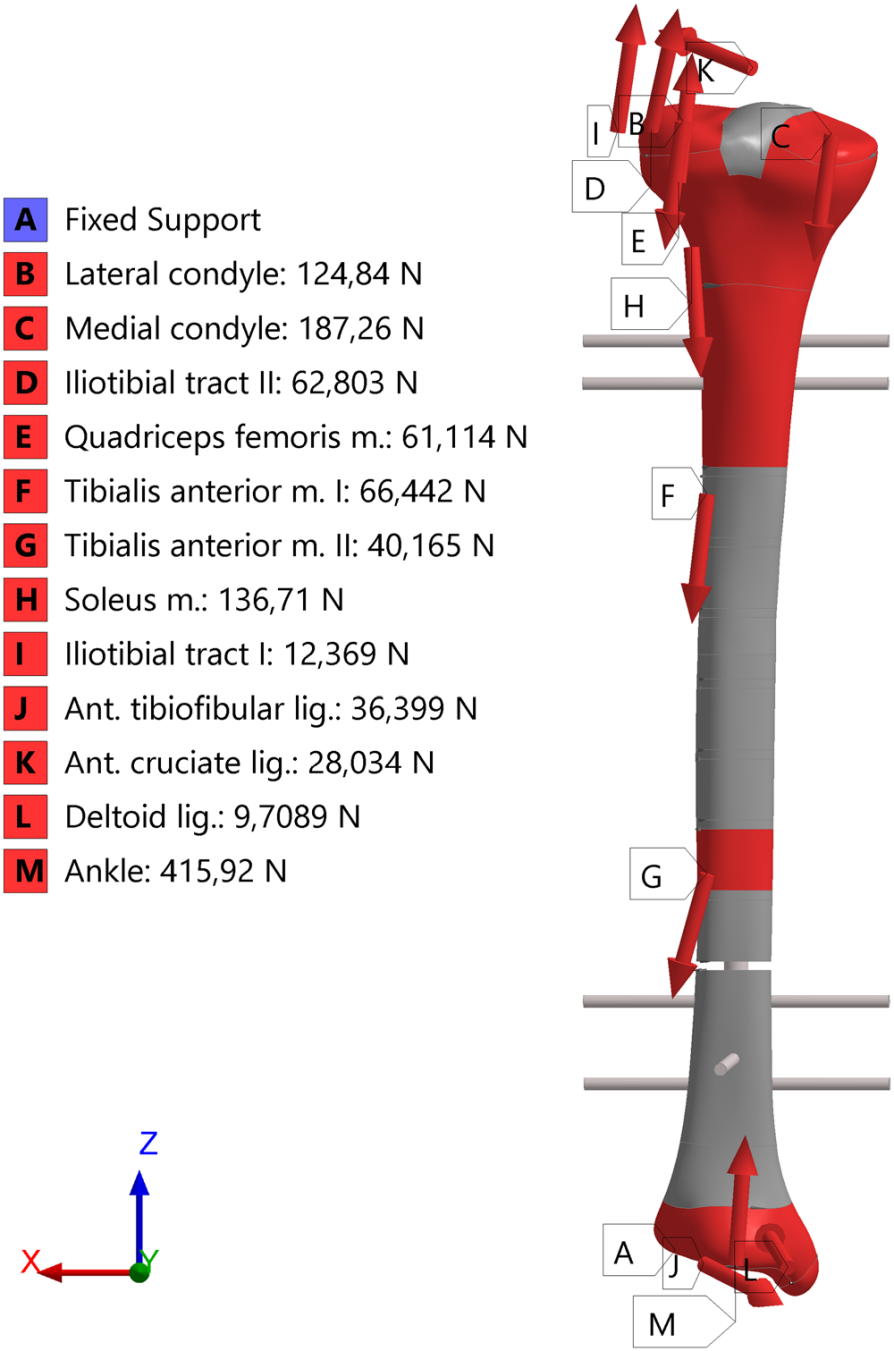
Graphical representation of the resultant forces applied to the model.

**Figure 4:**
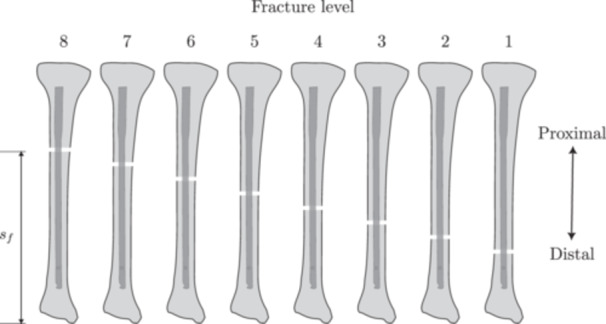
Eight tibial fracture sites modeled for subsequent finite element analysis. From left to right we have considered $s_{f}\in \{240,220,200,180,160,140,120,100\}$ in millimeters. The numbers on the top indicate the fracture level from 8 (proximal fracture) to 1 (distal fracture).

The geometry and dimensions of the intramedullary nail were obtained from the technical specifications provided by *Toride Implants* (Mogi Mirim, SP, Brazil). The device was inserted into the tibia in such a way that the entire nail was contained within the trabecular bone region. Two configurations were considered: (i) a clearance of 1 mm between the endosteal surface of the bone and the nail (i,e., the reamed canal was 1 mm larger than the nail), and (ii) no clearance. The fixation screws were modeled as cylindrical screws with diameter d=4 mm to avoid stress concentrations from screw threads in the comparative analysis. This simplification is appropriate as the study's focus is on the global structural behavior and load distribution of the entire construct, rather than on the localized stress concentrations in the screw threads, which are beyond the scope of this comparative analysis. Model features and geometric parameters are shown in Figures 1 and 2. The axial length of the tibia and the intramedullary nail are $L_{t}=380$ mm and $L_{n}=300\text{mm}$. The fracture was simulated using a bone osteotomy with a thickness of t=3mm. The distance $s_{f}$ is the distance from the plane Z = 0 (see the coordinate system in Figure 1) to the distal face of the fracture. This distance was considered a design variable in the subsequent analysis, as illustrated in Figure 1, 2, 3, 4, and was varied over the set $\;s_{f}\;\in \;\{240,\;220,\;200,\;180,\;160,\;140,\;120,\;100\}$ in millimeters.

The implant‐screw and bone‐screw interfaces were considered to have no gaps between components. Finally, the model was parametrized for several tibial fracture situations, from proximal to distal regions (see Figure 4).

### 2.2 Finite Element Model

The finite element model was developed in ANSYS Workbench employing 10‐node quadratic tetrahedral elements for all components. Bone is known to be a complex multi‐scale structure with heterogeneous, anisotropic, age‐dependent, and patient‐specific mechanical properties. Specifically, the cortical bone was simulated as a linear isotropic elastic material. An ideal elastoplastic material model (with no hardening after reaching the yield strength [21]) was used to account for possible local collapse of trabeculae. Finally, the metallic (ASTM F136) intramedullary nail and the fixation interlocking screws were simulated using an elastoplastic material model incorporating isotropic linear hardening behavior. The corresponding material parameters used for each constitutive model are shown in Table 1.

**Table 1:** Engineering constants for the material models utilized in the analyses.

	E (GPa)	ν	$\sigma_{y}$ (MPa)	$E_{t}$ (GPa)	Ref.
Interlocking screws	110.0	0.34	828.0	1.25	[27]
IMN	110.0	0.34	828.0	1.25	[27]
Trabecular bone	5.0	0.30	50.0	0.0	[21, 28]
Cortical bone	17.0	0.3	—	—	[21, 28]

**Figure 5:**
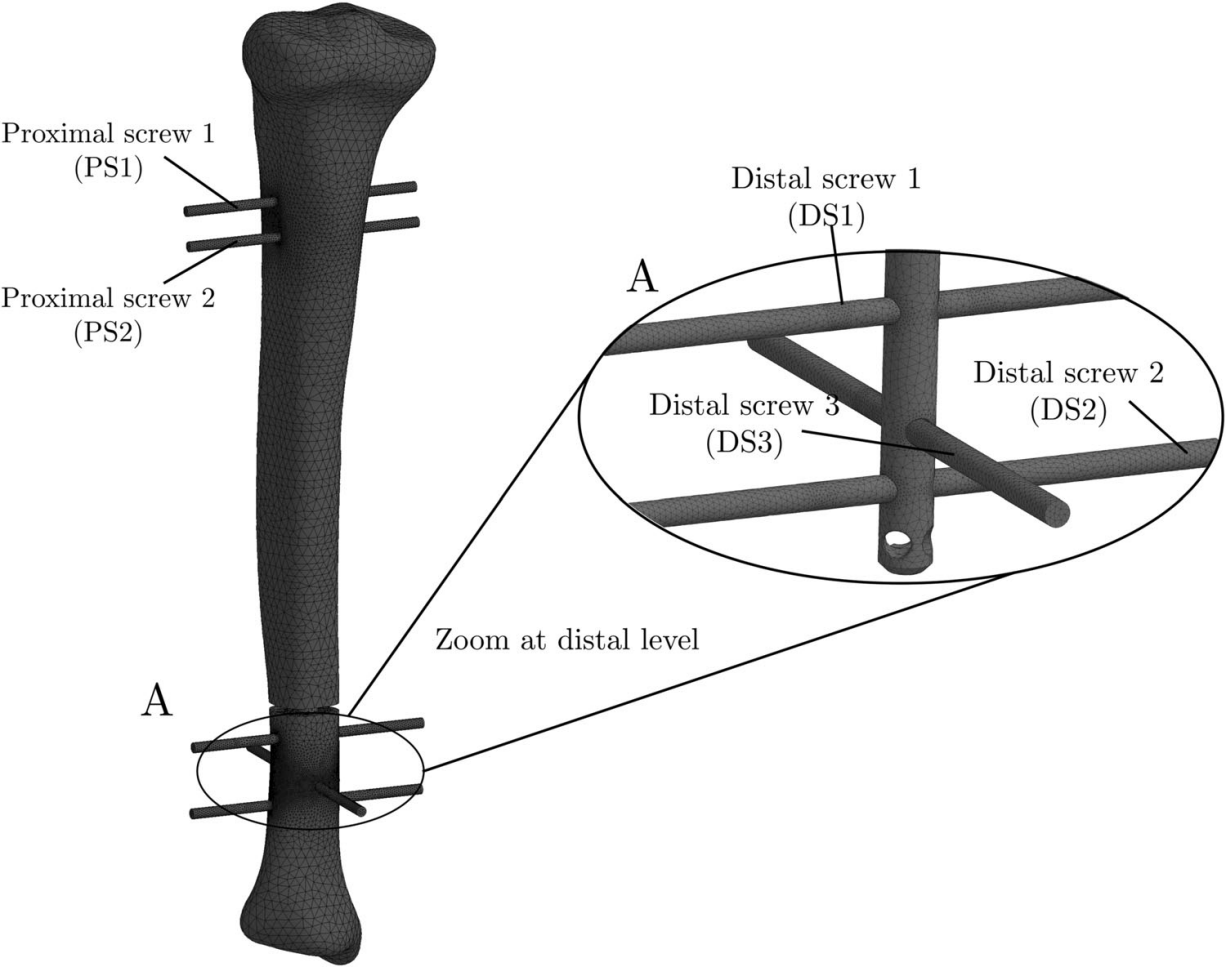
Finite element mesh showing the distal portion of the model, highlighting the interlocking screws DS1, DS2, and DS3).

The mesh was discretized using different element sizes for each component and region to optimize computational cost. A mesh convergence analysis was performed for all configurations, including cases with and without the interlocking DS3 screw and with and without clearance between the nail and the endosteal surface of the bone. The final models comprised between 230,245 and 982,775 tetrahedral elements, corresponding to the configurations without and with clearance, respectively. The interlocking screws were modeled with 1 mm elements, while the bone and the nail were discretized using 4 mm and 2 mm elements, respectively. A refined mesh of 0.5 mm elements was employed at the bone‐screw and implant‐screw contact interfaces. For the model without clearance between the endosteal surface of the bone and the nail, a refined mesh of 1.5 mm elements was adopted, whereas for the model with clearance, the refined mesh was 0.5 mm. The finite element mesh of the model without clearance is illustrated in Figure 5.

The interface mechanical conditions were defined according to the geometrical and functional characteristics of the modeled components. A no‐slip condition (displacement continuity) was imposed between the trabecular and cortical bone. For the interaction with the intramedullary nail, frictionless contact was defined at the interface with the trabecular bone and the interlocking screws. In contrast, a no‐slip condition was assigned between the cortical bone and the interlocking screws. This modeling choice is supported by clinical evidence indicating that the cortical bone is responsible for transmitting the majority of the mechanical loads imposed by the implant. As a result, no contact was assumed between the trabecular bone and the interlocking screws.

The values and position of contact, muscular, and ligament forces applied to the model were obtained from literature data [22, 23], considering a total body weight of 800 N. Nevertheless, to simulate the early postoperative phase, it was assumed that only 20% of the body weight is effectively transmitted through the limb, leading to the reduced loading conditions presented in Table 2. The condylar contact forces were distributed with 60% applied medially and 40% laterally, in agreement with the biomechanical findings reported in [22, 24]. The distal end of the tibia was constrained by fully fixing the distal articular surface [22, 25].

**Table 2:** Components of the applied force on each tibial condyle and the ligaments, considering 20% of total load of body weight. Duda et al. [22].

	Forces [N]			Attachment [mm]		
	*F*_*X*_	*F*_*Y*_	*F*_*Z*_	*X*	*Y*	*Z*
Iliotibial tract I	−1.70	−1.76	12.26	−19.80	39.10	369.90
Iliotibial tract II	−19.48	−12.88	58.30	−27.10	28.20	370.50
Quadriceps femoris m.	2.72	−6.56	60.70	34.80	19.30	353.40
Tibialis anterior m. I	3.44	7.74	−65.54	−4.90	9.90	251.80
Tibialis anterior m. II	5.18	10.72	−38.36	−7.60	9.90	127.90
Soleus m.	−12.62	−9.42	−135.80	−7.40	15.00	332.20
Ant. tibiofibular lig.	−26.48	−22.24	−11.36	−7.30	11.10	0.90
Ant. cruciate lig.	17.50	20.30	8.22	10.30	−5.00	390.50
Deltoid lig.	8.98	1.94	3.14	−1.50	−16.50	−0.30
Medial condyle	27.88	25.79	−183.37	−30.38	−30.79	369.07
Lateral condyle	18.58	17.19	−122.25	−25.02	17.81	373.09
Ankle	−24.00	−30.88	414.08	0.80	0.60	0.40

## 3 Results

This section presents the numerical results obtained from simulations performed for each distal fracture configuration (see Figure 4) considering cases with and without the inclusion of the interlocking screw DS3 (the 5th interlocking screw) and with and without clearance between the nail and the endosteal surface of the bone.

**Figure 6:**
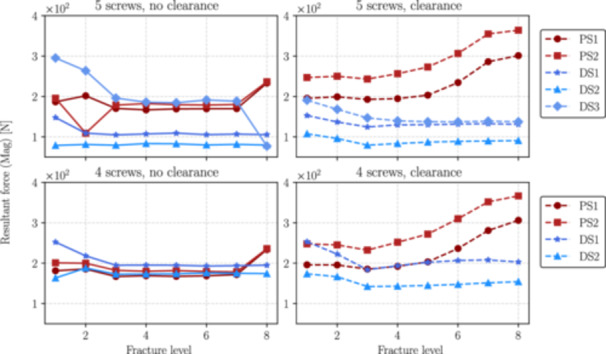
Magnitude of the resultant forces by the level of fracture (from distal to proximal) for all interlocking screws with and without considering the interlocking DS3.

**Figure 7:**
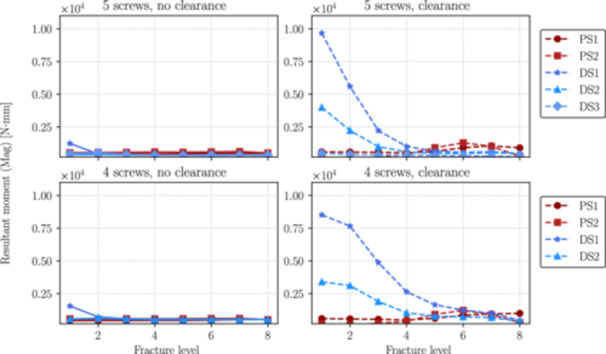
Magnitude of the resultant moment by the level of fracture (from distal to proximal) for all interlocking screws with and without considering the interlocking DS3.

**Figure 8:**
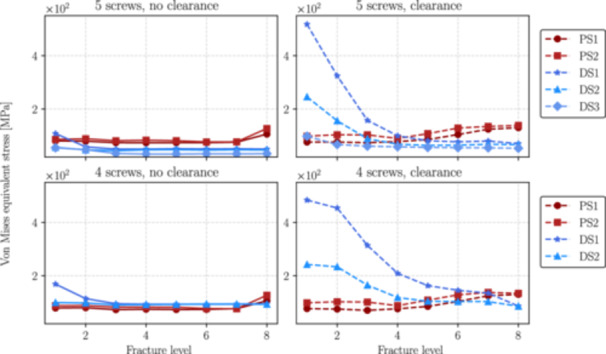
Maximum value of the von Mises equivalent stress by the level of fracture (from distal to proximal) for all interlocking screws with and without considering the interlocking DS3.

Figures 6, 7, and 8 show the magnitudes of the reaction forces, reaction moments, and maximum von Mises equivalent stress on all interlocking screws due to their contact with the intramedullary nail. The results are presented both with and without the inclusion of the interlocking screw DS3, and with and without a 1 mm clearance between the nail and the reamed canal. The maximum stresses were captured from the tensile region of screws subjected to flexure loads (corresponding to the areas with the highest principal stress values), which are prone to fatigue nucleation and fracture propagation. The horizontal axis in all figures represents the fracture level, where *N* = 1 corresponds to the most distal fracture $s_{f}=100\;\text{mm}$) and *N* = 8 corresponds to the most proximal fracture ($s_{f}=240\text{mm}$).

It is noteworthy that screw DS1 (see Figure 1) is the most loaded among all and therefore, the most susceptible to failure.

**Figure 9:**
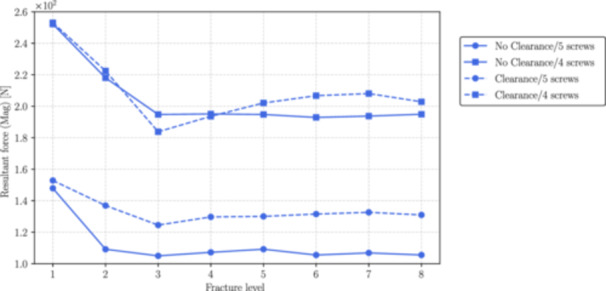
Magnitude of the resultant force at interlocking DS1 with and without the inclusion of interlocking DS3 by the level of fracture (from distal to proximal).

**Figure 10:**
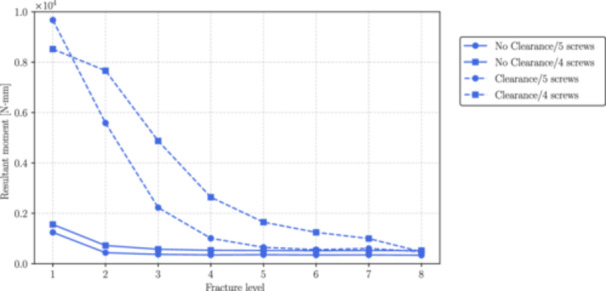
Magnitude of the resultant moment at interlocking DS1 with and without the inclusion of interlocking DS3 by the level of fracture (from distal to proximal).

**Figure 11:**
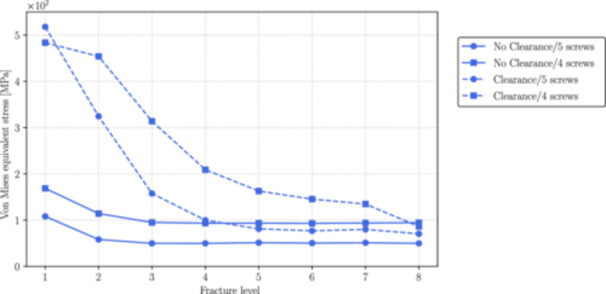
Maximum value of the von Mises equivalent stress at interlocking DS1 with and without the inclusion of interlocking DS3 by the level of fracture (from distal to proximal).

**Figure 12:**
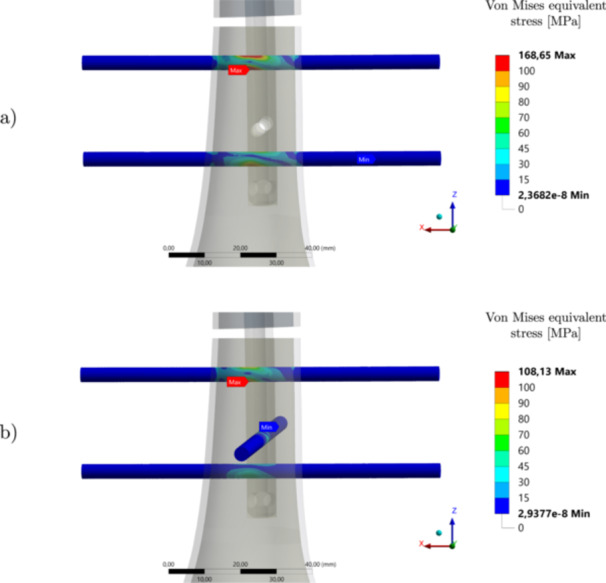
Von Mises equivalent stress field for the distal interlocking screws for both configurations. (a) without and (b) with the inclusion of the interlocking DS3. The figure showcases the critical case of fracture level 1 (most distal) and no clearance.

Figures 9, 10, and 11 compare the magnitude of the resultant force, resultant moment and maximum von Mises stress, respectively, acting on DS1 due to the contact with the intramedullary nail for all configurations. Again, the maximum von Mises stress displayed is that found in the tensile region of DS1, which is most prone to fatigue failure. The critical case (fracture level 1) is illustrated in Figure 12, where the von Mises equivalent stress field is displayed over the distal interlocking screws.

**Figure 13:**
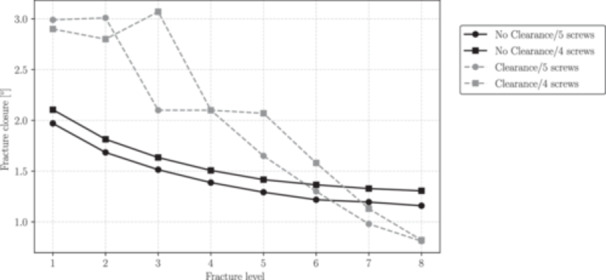
Fracture closure (in degrees) for each fracture level. The fracture closure is defined as the relative angle between the two fracture surfaces at the end of the performed analyses.

A measure of the stability provided by the nailing fixture was defined by computing the magnitude of the relative angle movement $\left|\theta_{\text{r}}\right|$ between the fracture surfaces after loading. Figure 13 shows how this angle varies depending on the fracture level for both configurations, with and without the fifth screw. To determine this angle, the rotation of each face relative to its centroid on the *X*, *Y* and *Z* axes was calculated. Thus, the relative angle is defined as:

(1) $$\left|\theta_{\text{r}}|=|\theta_{top}|-|\theta_{\text{bottom}}\right|$$

where

(2) $$|\theta_{i}|=\sqrt{\theta_{X,i}^{2}+\theta_{Y,i}^{2}+\theta_{Z,i}^{2}},\;i=\text{top,}\;\text{bottom}.$$

## 4 Discussion

All interlocking screws in the system can be visualized as a beam supported at its ends by the elastic cortical bone with a central load (force and moment) produced by the intramedullary nail. The contact between the interlocking screws and the trabecular bone is neglected, as the cancellous bone undergoes compaction after a few physiological load cycles, effectively eliminating its contribution to load‐bearing.

Due to the geometric characteristics of the tibia, the curvature of the intramedullary nail, and the distribution of the knee load in the condyles (60% medial and 40% lateral), the reaction forces and moments play a critical role in the mechanical equilibrium of the interlocking screws. The effects of these generalized forces are particularly pronounced as the location of the fracture changes distally along the tibia.

As can be seen in Figure 9, without considering the 1 mm clearance, the force on the interlocking screw DS1 due to its contact with the intramedullary nail remains relatively constant in the interval between fracture level 3 to 8, increasing when the fracture reaches distal tibia. Also, the inclusion of the fifth distal interlocking screw (DS3) reduces the magnitude of the resultant force by an average of 45%. When considering the 1 mm clearance between the nail and the endosteal surface of the trabecular bone, the reduction on the resultant force is still significant but less pronounced.

Similarly in Figure 10, without considering the 1 mm clearance, the moment on DS1 screw produced by the nail, mostly flexural, is relatively low at fracture levels range 3 to 8, but increases progressively as the fracture approaches to the distal region of the tibia. Also, the inclusion of the fifth distal interlocking screw (DS3) reduces the magnitude of the resultant moment at the interface with the intramedullary nail by an average of 33%. In the presence of clearance, the resultant moment on DS1 increases significantly, as the nail has reduced contact with the surrounding canal and is supported primarily by the screws.

With clearance between the nail and the canal, the moment transmitted to DS1 (see Figures 9, 10) largely governs the von Mises stress response of the screw, as reflected by the similar patterns in the moment and stress graphs (see Figure 11). This stress measure is crucial to assess risk of static/fatigue interlocking screw failure. As shown in Figure 11 in the case of no clearance, the maximum von Mises stress at the critical point of the screw remains relatively constant from fracture levels 8 to 3 but increases significantly as the fracture shifts from fracture level 3 to 1. With clearance, the maximum von Mises stress exhibits a nonlinear increase from the most proximal to most distal fracture levels. For the case without 1 mm clearance, Figure 11 shows that, the inclusion of the fifth interlocking screw (DS3) reduces by an average of 45% the stress levels in the interlocking screw DS1. This reduction is particularly critical for fracture levels between 1 and 2 ($100\;<\;s_{f}\;<120$). For the case with 1 mm clearance, except for the fracture level 1, the inclusion of the fifth interlocking screw (DS3) reduces by an average of 36% the stress levels in the interlocking screw DS1. This difference in stress levels, when considering a full body weight load, can lead to plastic deformations that may complicate the removal of the screw after surgery and hinder fracture stabilization, which is a critical factor in determining the success of postoperative outcomes.

It is also important to note that stress levels increase substantially when a 1 mm gap between the nail and the reamed canal is considered. For instance, as shown in Figure 11, for fracture 1, the stress rises by 185% from the no‐clearance case to the clearance case with 4 screws, and by 380% from the no‐clearance case to the clearance case with 5 screws.

For the case without clearance, the reduction in the contact reaction moment between the nail and DS1 is clearly more pronounced than the reduction in the reaction moment due to the insertion of the fifth screw (DS3). Therefore, the difference in von Mises stress caused by DS3 can be primarily attributed to reaction forces rather than moments. This is not the case when a 1 mm gap between the nail and the reamed canal is considered. In this situation, the reduction in moment is more pronounced and follows the same pattern as the von Mises stress, indicating that the stress difference is mainly due to reaction moments rather than reaction forces.

For the specific present load case that considers 20% of the body weight, the developed von Mises stresses for the no‐clearance case are much lower than the titanium (ASTM F136) yield stress and the use of a fifth screw would not be necessary. However, for the case when a 1 mm gap between the nail and the reamed canal is considered, the stresses increase substantially, and in critical cases in which the patient is subjected to a full body weight, or even partially (e.g., 50% or 75% body weight) the insertion of a fifth screw can maintain the screws within a safe elastic deformation regime. The von Mises stresses at fracture levels 1 and 8 in the clearance case show a nonintuitive behavior: these are the only levels where the stresses with the inclusion of the fifth screw are higher (level 1) or similar (level 8) compared to using only four screws. This arises from an abrupt change in contact behavior between the nail and the endosteal surface. With only two screws in the metaphyseal region, the area is less stable, allowing the nail to shift into the gap and make contact with the bone. This contact redistributes forces among the screws, stabilizing the stress behavior between levels 1 and 2. In contrast, three screws create a stable metaphysis, preventing the nail from advancing into the gap and redistributing the load. Consequently, the stress in the third screw continues to increase from level 2 to 1. To further clarify this behavior, we present additional simulation results of the contact pressure and sliding distance between the nail and the endosteal surface in Section A of the Supporting Material.

The relative angle between the fracture surfaces provides a quantitative measure of fracture stabilization. As shown in Figure 13, the addition of a fifth screw brings no significant changes in fracture stabilization when no clearance is considered. The behavior of fracture closure across fracture levels becomes more complex when clearance is considered, as the altered load transfer between the nail and the canal modifies the balance of forces sustained by the screws and surrounding bone. This suggests that the inclusion of a third distal transverse screw does not offer clear advantages in terms of stabilization and stiffness at the fracture site. These findings are aligned with experimental studies on fractured tibiae in regions similar to fracture level 1, which indicate that the use of three distal screws does not significantly enhance the system's stiffness [19].

Despite the insights gained through the present numerical analysis, a set of limitations should be addressed in future research. One of the key assumptions in the model is the idealized treatment of bone properties for both cortical and trabecular bone, which do not fully capture the inherent heterogeneity of the tibia. Specifically, the bone's modulus of elasticity is treated as constant, while in reality, it varies significantly with bone density, which can differ greatly between individuals. Older patients, for example, tend to have less dense bone structures, potentially leading to altered load distributions that the current model does not account for.

The loadings analyzed in this study are quasi‐static, assuming very slow loading rates. However, this does not reflect the dynamic nature of loads applied during daily activities, where forces fluctuate and can increase significantly. Dynamic loading studies are therefore essential to identify critical load cases, as these conditions could amplify the applied stresses. Also, only a transverse fracture was considered. Other fracture types, such as oblique or comminuted, may produce different equilibrium states and could therefore lead to alternative conclusions regarding the influence of fracture level on screw integrity. Additionally, a fatigue analysis is crucial because the applied loads are cyclic in nature, and the screws, with their complex geometries and numerous stress concentrators, may fail at much lower stresses than their yield strength under such conditions. A future study should focus on determining the optimal number of distal screws required to keep the system's stress levels below the endurance limit (infinite life under cyclic loading).

## 5 Conclusion

The numerical results indicate that the addition of a fifth distal screw (DS3) to the intramedullary nail configuration significantly improves the design's safety, particularly by reducing von Mises stress, especially in distal fractures. Stability, on the other hand, assessed through the relative movement of the fracture surfaces, exhibits less pronounced improvement compared to stress reduction. In addition to evaluating the inclusion of a third distal screw, the influence of a 1 mm clearance between the nail diameter and the reamed canal was also assessed. The stresses in the interlocking screws are clearly higher in the clearance case, which is expected, since in the absence of nail–bone contact the screws are responsible for maintaining stability. With clearance, the stress in the critical DS1 screw increases progressively as the fracture becomes more distal, whereas in the no‐clearance case, the stresses in this screw remain nearly constant until the fracture level 2. The difference in stress levels between the clearance and no‐clearance cases suggests that minimizing reaming during surgery may benefit screw integrity.

Thus, mechanical benefits must be weighed against the clinical implications. As highlighted in the literature, the addition of screws increases surgical complexity, prolongs operative time, and also raises patient exposure to radiation during fluoroscopic imaging, a critical factor that must be carefully managed. Also, it is well known that under‐reaming carries clinical risks, including implant incarceration and iatrogenic fractures [18, 26].

In summary, while including the fifth screw (DS3) improves certain mechanical aspects, it may not substantially enhance stabilization or stiffness across all fracture levels. Although the results indicate that reducing clearance between the nail and the endosteal surface lowers stresses in the interlocking screws, the clinical consequences of such reaming decisions must also be considered. For design purposes, we recommend that intramedullary nail systems be designed based on the critical case, that is, assuming a uniform clearance between the nail and the canal. In this scenario, most of the load is transmitted through the screws rather than shared with the endosteal surface via contact with the nail.

## Ethics Statement

The authors have nothing to report.

## Conflicts of Interest

Tóride S. Celegatti, one of the authors of this study, is the CEO of Toride IND. COM. LTDA, the company that provided the implant geometry used in this study. While Tóride S. Celegatti contributed to the study design and manuscript preparation, we affirm the integrity, independence, and objectivity of the research process and its findings. Additionally, it is important to disclose that Eduardo A. Fancello, José L. M. Thiesen, Carlos R. M. Roesler, and Rafael Prado hold research grants from the aforementioned company for product development, while Kodi E. Kojima serves as a medical consultant.

## Supporting information

Supporting Material Thiesen et al 2025.

## Data Availability

The authors have nothing to report.
