# Supplementary material for: Load Transfer in Tibial Intramedullary Nailing: Effects of Fracture Level, Screw Configuration and Nail‐Canal Clearance
Source: J Orthop Res. 2025 Dec 16;44(1):e70102. doi: 10.1002/jor.70102 (PMC12706723; doi:10.1002/jor.70102)
Supplement: Supplementary file 1 — Supporting Material Thiesen et al 2025. [file JOR-44-0-s001.docx]

Supplementary Materials - The Effect of Fracture Level and Number of Screws on the Overloading of Distal Locking Screws for Tibial Intramedullary Nailing

**Thiesen**, Jose´ L. M.^a,b^, **Prado**, Rafael^a,b^, **Kojima**, Kodi E.^d^, **Celegatti**, To´ride S.^c^, **Mendonc¸a**, Paulo de Tarso R.^a^, **Roesler**, Carlos R. M.^b^, **Fancello**, Eduardo _A._a,b,∗

*^a^GRANTE, Department of Mechanical Engineering, Federal University of Santa Catarina, Floriano´polis, Santa Catarina, Brazil*

*^b^Laboratory of Biomechanical Engineering (LEBm), Federal University of Santa*

*Catarina, Floriano´polis, Santa Catarina, Brazil*

*^c^Toride Implants Industry and Commmerce, Parque das Empresas, Mogi Mirim, Sa˜o Paulo, Brazil*

*^d^Institute of Orthopedie and Traumatology (IOT/USP), University of Sa˜o Paulo, Sa˜o Paulo, Sa˜o*

*Paulo, Brazil*



**1. Section A**

Figures 10 and 11 of the main text present results for the resultant contact moment at the first distal screw (DS1) and the von Mises equivalent stress at its critical tensile region. This section of the supplementary material aims to explain the non-intuitive change in the load-transfer regime observed between fracture levels 2 and 1, in the case of 1 mm clearance.

Although the addition of a fifth screw reduces moments and stresses at fracture levels 2 to 8, the opposite trend is observed at level 1, where the stresses increase relative to the four-screw case.

Figures [1](#_e0nw3uq1b7pc)a–b) of this supplementary material illustrate the contact pressure distribution between the nail and the endosteal surface for the clearance/4-screw configuration with a fracture at level 2 and 1. When the fracture occurs at level 1, the contact area between the nail and the bone wall increases, providing greater structural support around the fracture site (highlighted by the red arrows). This



^∗^Corresponding author. Email: [efancello@gmail.com](mailto:efancello@gmail.com)

*Preprint to Journal of Orthopaedic Research September 8, 2025*

additional support explains the stabilization of moments and stresses observed in the screws seen at Figs. 10 and 11 of the main text.



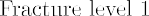

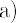


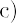


Figure 1: a) and b) Nail-to-bone contact pressure for the case considering the reaming clearance of 1 mm and 4 interlocking screws (without DS3) in the intramedullary nail system. d) and e) Nail-to- bone contact pressure for the case considering the reaming clearance of 1 mm and 5 interlocking screws (with DS3) in the intramedullary nail system.

In contrast, Figs. [1](#_e0nw3uq1b7pc)c–d) display the same quantities for the clearance/5-screw configuration. When the fracture occurs at level 1, the nail loses contact with the bone wall distal to the fracture (as highlighted by the red arrow), resulting in reduced structural support and, consequently, increased moments and von Mises stresses in DS1. This increase is clearly seen in Figs. 10 and 11 of the main text between fracture levels 1 and 2.

2
